# Structure and Dynamics of Minke Whale Surfacing Patterns in the Gulf of St. Lawrence, Canada

**DOI:** 10.1371/journal.pone.0126396

**Published:** 2015-05-13

**Authors:** Fredrik Christiansen, Ned M. Lynas, David Lusseau, Ursula Tscherter

**Affiliations:** 1 Centre for Integrative Ecology, School of Life and Environmental Sciences, Deakin University, Warrnambool, Victoria, Australia; 2 Foundation for Marine Environment Research (ORES), Basel, Switzerland; 3 Ocean Research and Education Society (ORES), Les Bergeronnes, Quebec, Canada; 4 Institute of Biological and Environmental Sciences and Institute of Marine Alliance for Science and Technology for Scotland, University of Aberdeen, Aberdeen, United Kingdom; New York Institute of Technology College of Osteopathic Medicine, UNITED STATES

## Abstract

Animal behavioral patterns can help us understand physiological and ecological constraints on animals and its influence on fitness. The surfacing patterns of aquatic air-breathing mammals constitute a behavioral pattern that has evolved as a trade-off between the need to replenish oxygen stores at the surface and the need to conduct other activities underwater. This study aims to better understand the surfacing pattern of a marine top predator, the minke whale (*Balaenoptera acutorostrata*), by investigating how their dive duration and surfacing pattern changes across their activity range. Activities were classified into resting, traveling, surface feeding and foraging at depth. For each activity, we classified dives into short and long dives and then estimated the temporal dependence between dive types. We found that minke whales modified their surfacing pattern in an activity-specific manner, both by changing the expression of their dives (i.e. density distribution) and the temporal dependence (transition probability) between dive types. As the depth of the prey layer increased between activities, the surfacing pattern of foraging whales became increasingly structured, going from a pattern dominated by long dives, when feeding at the surface, to a pattern where isolated long dives were followed by an increasing number of breaths (i.e. short dives), when the whale was foraging at depth. A similar shift in surfacing pattern occurred when prey handling time (inferred from surface corralling maneuvers) increased for surface feeding whales. The surfacing pattern also differed between feeding and non-feeding whales. Resting whales did not structure their surfacing pattern, while traveling whales did, possibly as a way to minimize cost of transport. Our results also suggest that minke whales might balance their oxygen level over multiple, rather than single, dive cycles.

## Introduction

Understanding the causal factors that determine how individual animals alter their behaviors, the underlying process of behavioral patterns, is a central question in ecology, physiology and neurology [1–3]. Studies of animal behavioral patterns can help us understand both physiological [3] and ecological [4] constraints on animals, and the potential consequences such constraints can have on population dynamics and conservation [5–7] by influencing individual fitness [8].

Behavioral patterns have evolved to optimize individual fitness over time [9]. Because behavioral patterns are determined by internal variables related to the individual (its motivational state) and external variables related to the environment (environmental state) [1,10,11], different behavioral patterns will be optimal during different conditions. The surfacing patterns of aquatic mammals have evolved as a trade-off between the need to replenish oxygen stores at the surface and the need to conduct other activities underwater [12–15]. As for behavioral patterns in general, both internal (e.g. body size, motivational state) and external variables (e.g. depth, prey density, predation risk) will determine which activity an animal engage in [16–19], which in turn will determine its surfacing pattern. Thus, an animal’s surfacing pattern reflects physiological constraints, given the animal’s short term “goals” (e.g. feeding or traveling) [20].

The dives of marine mammals (the time elapsed between two consecutive breaths) can generally be divided into relatively longer dives, during which the animal engage in some activity (e.g. foraging or traveling), and relatively shorter dives, when the animal replenishes its oxygen stores by taking a series of deep breaths in relatively rapid succession [21–23]. In this paper we will refer to these two dive types as long dives and short dives, respectively. Generally, as the duration of the long dive increases, the number of subsequent breaths, and hence short dives, increases as well to maintain sufficient oxygen stores [12,20,24–27]. Since the oxygen extraction efficiency decreases curvilinearly through a surfacing bout (a series of consecutive breaths/short dives between two long dives) [12,28], there is often a non-linear relationship between dive duration and the number of breaths/short dives [25,27,29,30]. During foraging, such non-linear relationships can also result from animals trying to minimize their time spent at the surface (i.e. the number of short dives) to increase feeding rates [29].

In this study we aim to better understand the surfacing pattern of the minke whale (*Balaenoptera acutorostrata*), a marine top predator, in the St. Lawrence estuary, Canada, a summer feeding ground [31]. The area lies within a marine protected area, the Saguenay—St. Lawrence Marine Park. Behavioral data have been collected from minke whales in this area for nearly two decades, with previous studies showing that the behavior of minke whales in this area can be divided into six distinct activities based on the horizontal movement pattern of the animals at the surface in combination with other behavioral cues [32]. Four of these activities relate to feeding, and can be further divided into surface feeding (two tactics), near-surface foraging and deep foraging, while the remaining two non-feeding activities can be separated into traveling and resting.

Our hypothesis is that each activity state will have its own distinct surfacing pattern. Starting with resting, there are no obvious benefits for minke whales, in terms of reducing energy expenditure, to structure their surfacing pattern and we would therefore expect their surfacing pattern to be random. When traveling, marine mammals generally minimize their cost of transport (COT) by diving to a few m depth where they are able to avoid the increasing drag created by the surface turbulence [33]. Since longer dives allow the animals to stay longer at this preferred depth, minke whales should benefit from structuring their surfacing pattern in a way that increases the duration of their long dives, while still staying within their aerobic dive limit (ADL) [18]. When feeding, the depth of the prey layer will influence the transit time (the time spent swimming vertically to and from the prey layer), and thus the time available for foraging, during a long dive. During surface feeding, when the prey is at the surface, transit time is zero, and the surfacing pattern should reflect prey handling time (the capture, processing and swallowing the prey once located) alone. During near-surface foraging and deep foraging, the depth of the prey layer, and hence transit time, will increase [12,13]. To compensate for this, baleen whales generally increase the number of feeding lunges during a dive as the depth of the prey layer increases [27]. As a result, the time spent at depth, and hence the overall dive duration, will increase with the depth of the prey layer [27]. The physiological constraint of having to surface to breath will result in a trade-off between foraging time (i.e. dive duration) and time spent (i.e. number of breaths) at the surface to replenishing oxygen stores, with the applied surfacing pattern being the one that maximizes the net energy gain per unit time [12,34,35]. Thus as the prey depth increases between surface feeding, near-surface foraging and deep foraging, we expect the surfacing pattern of minke whales to become increasingly structured, with an increasing number of breaths (i.e. short dives) needed between long dives, as a result of increasing physiological constraints.

We test these hypotheses using minke whales as an example. First, we use a quantitative approach to distinguish between dive types and to compare the expression of the different dive types (i.e. their density distribution) between activity states. We then estimate the temporal dependence between dive types both within and between activity states, to investigate the surfacing pattern of minke whales between activities. We start at a smaller temporal scale (a single dive cycle) and then extend the temporal scale by including an increasing number of dive cycles, to test over which temporal scale minke whales organize their surfacing patterns.

## Materials and Methods

### Data collection

All fieldwork was conducted under research permits (in years of requirement) issued to the Ocean Research and Education Society (ORES) by the Department of Fisheries and Oceans Canada (#QUE1990-2009) and Parks Canada (#R2002-2005, #SAGMP2006-2009). Following the guidelines of the Canadian Council of Animal Care in the category B level of invasiveness, the methods used are considered to have caused little or no discomfort or stress to the animals studied.

The behavior of minke whales was recorded by continuous individual focal follows in the study area between June and October from 1990 to 2009. Data was only collected in sea conditions of Beaufort three or less. For at least 30 min data was recorded by up to eight dedicated observers from a six m rigid hull inflatable boat staying within visual range of the animal. Individual whales were identified based on individually distinctive external characteristics. Minke whales can be identified on the basis of dorsal fin shape, body coloration patterns and distribution of scars [36].

To minimize disturbance, focal animals were always approached from the side and rear and this position was maintained during the follow at a minimum distance of 50–100m to give the animal full freedom of movement. Further, the speed of the research vessel was matched to the speed of the whale and rapid speed and course changes were avoided.

During focal follows the time to the second of each respiration surfacing, the heading of the animal, qualitative grade of arching (slight, regular or steep), and the position of the whale or research vessel at regular intervals (using a Global Positioning System (GPS)) was recorded. Surface feeding events (SFE), observations of minke whales engulfing prey at the surface (lunges and arcs) or right below (expanding footprints) [32] were registered, as well as surface corralling maneuvers (CM), which include lateral surfacings, rolls, head slaps and underwater exhales used prior to a SFE. Corralling maneuvers do not include food intake but are thought to aid in prey concentration and capture. Surface feeding events and CM may or may not include air intake. Samples with missed surfacing times and identities as well as of pairs and groups were excluded from the analysis, the latter to avoid potential behavioral biases resulting from social interactions.

Each focal follow was classified into a single activity state, based on visual observations of their surface behavior. If a whale changed it activity state during a follow, the follow was terminated. The different activity states for minke whales are defined in Table 1 (after [22,32,37]). Although minke whales are likely to be feeding along a continuum of depths, the lack of whale depth data forced us to classify foraging at depth into near-surface foraging (NSF) and deep foraging (DF), when whales were assumed to be foraging on prey located at depths above and below 50m, respectively. This crude threshold was based on previous research carried out in the area (N.M. Lynas pers. obs.), where a noticeable correlations was found between the degree of arching (Table 1) and the occurrence of prey at different depths. To verify this threshold, a fish-finding sonar (Lowrance LMS-334c iGPS) with a single frequency (200 kHz) Skimmer transducer was used to measure the depth of the assumed prey layer directly under the research vessel while following a whale during foraging. Linear models (LM) in R 2.14 [38] were fitted to the maximum and minimum depth of the prey layer recorded during SFT1, NSF and DF, to verify the depth of the prey layer during the different feeding activities. Because surface noise prevented use of the fish-finder immediately below the surface, visual observations of fish at the surface was used to record fish presence at the surface (minimum depth = 0).

**Table 1:** Definition of activity states of minke whales in the St. Lawrence, Canada.

Activity category	Activity state	Definition
Surface feeding	Surface feeding tactic I (SFT1)	Respiration surfacings and presence of SFE, but no CM.
	Surface feeding tactic II (SFT2)	Respiration surfacings and presence of both SFE and CM.
Foraging at depth	Near-surface foraging (NSF)	Respiration surfacings, tight J or O-shaped surface swimming patterns. Slight to regular dorsal arches. Shallow diving angles (< 30°).
	Deep foraging (DF)	Respiration surfacings, tight J or O-shaped surfaceswimming patterns. Strong dorsal arches. Steep diving angles (> 30°).
Non-feeding	Traveling (TRA)	Slow respiration surfacings with a straight swimming pattern. Very slight dorsal arches or none.
	Resting (REST)	Slow respiration surfacings with non-directional swimming pattern. Very slight dorsal arches or none.

SFE = Surface feeding event, CM = Corralling maneuver.

### Dive types—the behavioral expression

From the surfacing times that included air intake, minke whale inter-breath intervals (IBI) were calculated as the time elapsed between two consecutive breaths in a follow. To identify different dive types within the IBI data we fitted a Gaussian univariate mixture model to the density distribution of log-transformed IBI, using Expectation maximization (normalmixEM in R package mixtools). Different numbers of dive types were tested for each activity state. Based on the posterior probabilities of the best fitting mixture model, each IBI was then quantitatively classified as either a short dive or a long dive, using a threshold value of λ 0.5. A separate mixture model was used for each activity state to test if the behavioral expression (i.e. density distribution of IBI) varied between activities [39]. To test whether the expression of short dives varied depending on the duration of the long dives, linear mixed effects models (LME; lme in R package nlme) were used to test the relationship between the mean IBI of the short dives within a surfacing bout and the dive duration of the long dives, both before and after the bout.

### Surfacing patterns—the behavioral process

The surfacing breathing patterns (hereafter referred to as surfacing patterns) of minke whales were investigated by first investigating the temporal dependence between dive types (the behavioral process) [39], by estimating the transition probability between dive types [22,23,40]. We ran separate analyses for each activity state, to test if minke whales structure their surfacing pattern differently depending on their activity. The temporal dependence between dive types was estimated using a first-order Markov chain [41]. The time series of dive types (one for each follow) were first compiled into two-way contingency tables of preceding versus succeeding dive type [40,42]. Transition probabilities from preceding to succeeding dive type were then calculated [42]:

$$P_{ij}=\frac{a_{i}{}_{j}}{\sum_{j=1}^{n}a_{ij}},\sum_{j=1}^{n}P_{i}{}_{j}=1$$

where *i* is the preceding dive type, *j* is the succeeding dive type, *n* is the total number of dive types (i.e. two), *a*_*ij*_ is the number of transitions observed from dive type *i* to *j*, and *P*_*ij*_ is the transition probability from *i* to *j* in the Markov chain. To test if the estimated contingency tables (i.e. surfacing patterns) differed from a theoretical distribution (random), goodness of fit tests were performed using Pearson's chi-squared test.

The relationship between the number of short dives within a surfacing bout and the duration of the long dive, for different activity states, was investigated using Generalized Linear Models (GLM; glm in R package stats) and Generalized Linear Mixed Models (GLMM; glmer in R package lme4) with Poisson distribution (the number of short dives represents non-negative count data) and log link function (predicted values cannot be negative). The number of short dives, rather than the surfacing bout duration, was used as response variable since the former relates directly to the number of breaths, and hence energy expenditure of the animal [43,44]. The number of short dives in a bout was compared to the duration of both the preceding and the succeeding long dive, to find out whether minke whales prepare or recover from these dives [25,29,45]. Activity was added as an interaction term in the model to test if the relationship between number of short dives and dive duration differed between activity states. Both linear and non-linear (different polynomial) models were fitted to the data to test which best described the relationship between number of short dives and dive duration. To look for potential non-linear relationships outside the range possible for GLMs, a generalized additive model (GAM; gam in R package mgcv) with a thin plate regression spline smoother and a Poisson distribution and log link function was also fitted to the data. The best fitting model was selected using Akaike’s Information Criterion (AIC). The marginal coefficient of determination (*R* ^2^_m_), which represents the amount of variance explained by the fixed factors in the model, was also estimated for each model.

### Determining the temporal scale of surfacing patterns

To investigate if the surfacing pattern of minke whales varied at different temporal scales, we estimated the respiration rates (number of breaths min^-1^) of minke whales over different number of dive cycles, ranging from one to six. With the obtained estimates we then created boxplots to allow for visual examination of the distribution of values at different temporal scales (number of dive cycles).

### Model validation

The variance inflation factor (VIF; vif in R package car) was used to investigate collinearity (high correlation) between the explanatory variables in the model. An upper VIF threshold value of three was used to remove collinear variables, one at a time, until all VIF values were below three and no collinearity remained in the model [46]. For all models, model validation tests were run to identify potential violations of the assumptions of the given model. Scatter plots of residuals versus fitted values and residuals against each explanatory variable were used to test the assumption of equal variances (homogeneity) in the model. Normality of residuals was interpreted from Quantile-Quantile plots and from residual histograms [46]. We also looked for influential points and outliers using leverage and Cook’s distance. For the Poisson models, overdispersion was tested for each model by dividing the residual deviance with the residual degrees of freedom, with a ratio value above 1.5 indicating overdispersion (the mean of the variance is larger than the mean).

## Results

Minke whale behavioral data was collected on 324 days between June and October 1990 and 2009, resulting in 298 h of minke whale follow data. A total of 24 579 surfacings were recorded, of which 22 639 included air intake, 3236 SFE and 4215 CM. Altogether 489 follows were conducted (SFT1 = 105, SFT2 = 123, NSF = 74, DF = 50, TRA = 41, REST = 24, Mix = 72), of which the individual could be identified in 72% (352) of the follows, as one of 89 known individuals. The average follow duration was 37 min (SD = 23.9).

**Fig 1:**
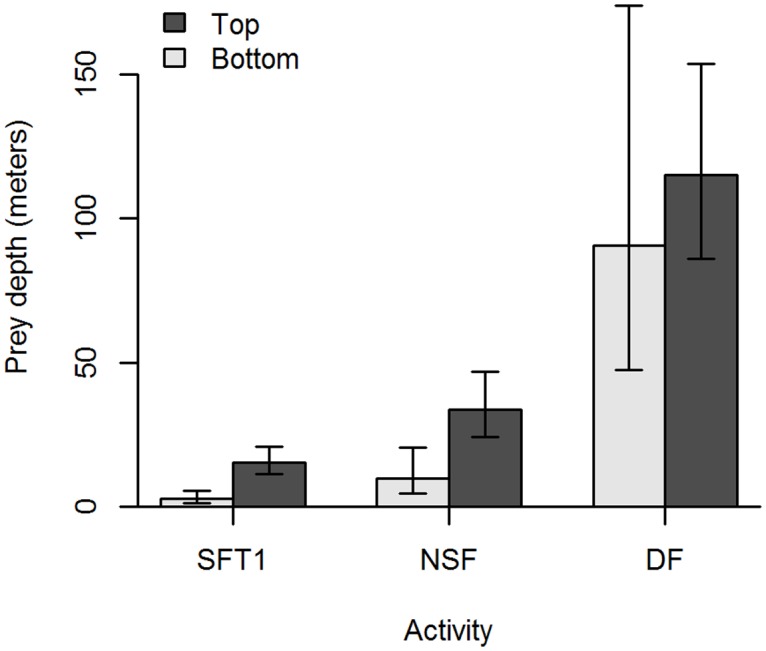
Back transformed prey layer depth recorded near minke whales during three different feeding activities. The depth at the top (dark grey) and bottom (light grey) of the prey layer is shown. SFT1 = surface feeding tactic I, NSF = near-surface foraging, DF = deep foraging. Error bars represent 95% confidence intervals. N = 21.

There was a significant difference in both the maximum (LM ANOVA: *F*_2,18_ = 51.1, *P* < 0.0001) and minimum (LM ANOVA: *F*_2,18_ = 30.9, *P* < 0.0001) prey depth between the different feeding activities (Fig 1), which supported the division of feeding activities into SFT1, NSF and DF. The models explained 85 and 77% of the variance (*R* ^2^) in maximum and minimum prey depth, respectively. Both models fulfilled the assumptions of the LM.

### Dive types—the behavioral expression

**Fig 2:**
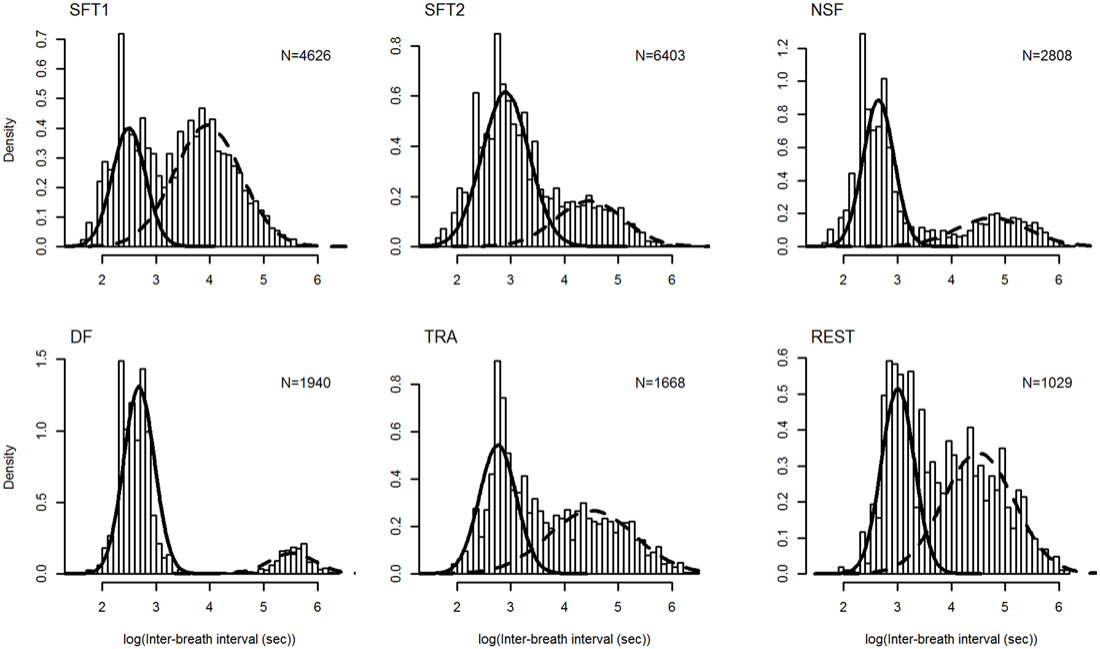
Density distribution of logged IBI for minke whales during different activity states. SFT1 = surface feeding tactic I, SFT2 = surface feeding tactic II, NSF = near-surface foraging, DF = deep foraging, TRA = traveling, REST = resting. The solid and dashed lines represent the density distributions of short dives and long dives, respectively. The sample size (N) for each activity state is shown at upper right corner of each subfigure.

The mixture models identified two distinct dive types (components) for each activity state, representing short dives and long dives (Fig 2). No activity showed support for more than two dive types. The means of the short dives were similar between activity states, although REST had slightly longer short dives (Table 2). The variation (SD) in duration of short dives was also similar between activity states, although relatively higher for SFT2 (Table 2). The means of the long dives varied considerably more, with NSF and DF having relatively longer dives compared to the other activities, which all had similar durations (Table 2). Apart from DF, the variation in duration of the long dives was high for all activity states (Table 2). The relative occurrence of the dive types (λ) varied substantially between activities (Table 2), with more than half the dives performed during non-feeding activities being long dives, whereas long dives made up a much smaller proportion of dives for feeding related activities, especially DF (Table 2). The only exception was SFT1, when whales performed mostly long dives. The overlap in density distribution between dive types (OVL) was similar for all activity states except NSF and DF which showed almost no overlap between dive types (Table 2).

**Table 2:** Model parameters from the univariate Gaussian mixture models for different activity states of minke whales.

Activity state	Short dive			Long dive			OVL	N
	mean	SD	λ	mean	SD	λ		
SFT1	2.46	0.32	0.33	3.88	0.66	0.67	0.14	4626
SFT2	2.84	0.46	0.71	4.41	0.63	0.29	0.15	6403
NSF	2.57	0.31	0.70	4.76	0.70	0.30	0.03	2808
DF	2.62	0.26	0.85	5.49	0.41	0.15	0.00	1940
TRA	2.85	0.32	0.44	4.36	0.84	0.56	0.17	1668
REST	3.01	0.30	0.39	4.35	0.72	0.61	0.17	1029

The mean dive durations (logged IBI (sec)), SDs and relative occurrences (λ) of the two dive types (short dive and long dive) identified for each activity (SFT1 = surface feeding tactic I, SFT2 = surface feeding tactic II, NSF = near-surface foraging, DF = deep foraging, TRA = traveling, REST = resting). Observe that λ constitutes only one parameter in the model, the second value of λ was obtained from 1-λ. OVL = Overlapping coefficient.

Consistent with the findings of the mixture models, the LME revealed a significant difference in the mean IBI of short dives (IBI_Short_) between activities (LME: mean(IBI_Short_)~Activity, ANOVA: *F*_5,393_ = 60.1, *P* < 0.0001). The variance attributed to the random effect (focal follows) was 5.36 and the residual variance was 17.04. There was no effect of dive duration on the mean IBI of short dives, and no interaction between dive duration and activity, suggesting that the expression of dive types was consistent within activity states.

### Surfacing patterns—the behavioral process

The transition probabilities between dive types, estimated from the Markov chains, revealed structured surfacing patterns for all activity states except REST, which had a random surfacing pattern (Table 3). The surfacing pattern of TRA whales was significantly structured, although the transition probabilities between dive types were close to 0.5 (Table 3). For the feeding related activities, the probability of changing from a short dive to a long dive was lowest for DF, followed by SFT2 and NSF (Table 3). The probability of performing two long dives in a sequence was almost non-existent for DF, whereas NSF and SFT2 had higher probabilities. In contrast, SFT1 showed the opposite pattern (Table 3), with whales being more likely to change from a short dive to a long dive, and also perform several long dives in a sequence. This pattern was consistent with the density distribution of SFT1 (Fig 2 and Table 2).

**Table 3:** Transition probabilities between dive types of minke whales for different activity states and test statistics from the Pearson’s chi-squared tests.

Activity state	Transition probability (*P*)				Pearson's chi-squared test		
	Short→Short	Short→Long	Long→Short	Long→Long	*X* ^2^	*P*-value	N
SFT1	0.44	0.56	0.35	0.65	36.53	<0.0001	4153
SFT2	0.74	0.26	0.81	0.19	30.20	<0.0001	5463
NSF	0.68	0.32	0.80	0.20	36.27	<0.0001	2716
DF	0.86	0.14	0.95	0.05	18.21	<0.0001	1890
TRA	0.54	0.46	0.46	0.54	9.27	0.0023	1619
REST	0.43	0.57	0.44	0.56	0.02	0.8988	999

SFT1 = surface feeding tactic I, SFT2 = surface feeding tactic II, NSF = near-surface foraging, DF = deep foraging, TRA = traveling, REST = resting. Short = Short dive, Long = Long dive. The degrees of freedom for all matrices were 1.

We found that the duration of the long dive prior to a surfacing bout was a better determinant of the number of breaths (i.e. short dives) in a bout, than the duration of the long dive succeeding a surfacing bout (Models 1 and 2 in Table 4). The most parsimonious GLMM included dive duration and activity, as well as an interaction term between dive duration and activity, as covariates (Model 7 in Table 4). The interaction term suggested that the effect of dive duration on the number of breaths (i.e. short dives) differed between activity states. Model fit was improved considerably by including focal follows as a random effect in the model. The random effect revealed that the number of breaths in a bout varied between follows by a variance of 0.23 (SD = 0.483). The dispersion parameter (φ) was 1.19, which indicated no overdispersion of the Poisson GLMM. There was no collinearity between the explanatory variables in the model (VIF < 3). The fixed effects of the model explained nearly half (49.2%) of the variance in the data.

**Table 4:** Model selection results of the GLMM to explain the number of short dives in a surfacing bout for minke whales.

Model	Fixed effects	RD	k	*R* ^2^_m_	φ	AIC	ΔAIC
1	Dive.pre	9803	3	0.048	1.18	9809	560
2	Dive.post	9977	3	0.029	1.20	9983	734
3	Activity	9855	7	0.407	1.25	9869	620
4	Dive.pre+Activity	9510	8	0.459	1.19	9526	277
5	Dive.pre×Activity	9388	13	0.466	1.20	9414	165
6	(Dive.pre+Dive.pre^2^)×Activity	9226	19	0.490	1.19	9264	15
7	(Dive.pre+Dive.pre^2^+Dive.pre^3^)×Activity	9199	25	0.492	1.19	9249	0

Dive.pre = duration of long dive preceding a surfacing bout, Dive.post = duration of long dive succeeding a surfacing bout, RD = residual deviance, k = number of parameters, *R* ^2^_m_ = the marginal coefficient of determination (the variance explained by the fixed effects), φ = dispersion parameter (deviance/(N-k), where N is the sample size). All models included focal follow as a random effect. N = 6901.

**Fig 3:**
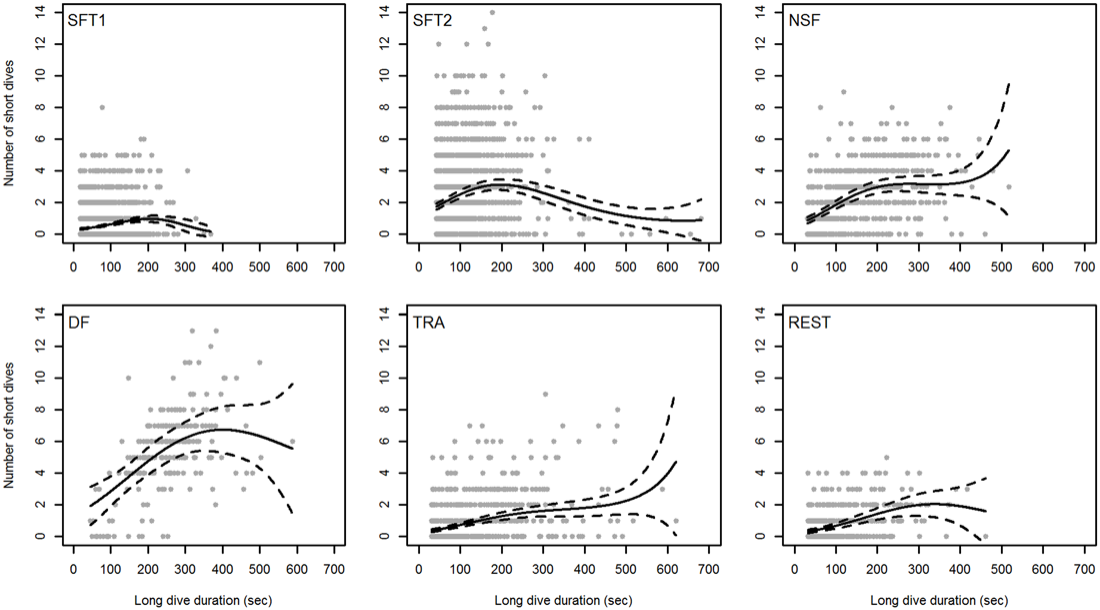
Back transformed number of short dives in a surfacing bout as a function of the dive duration of the preceding long dive for minke whales during different activity states. SFT1 = surface feeding tactic I, SFT2 = surface feeding tactic II, NSF = near-surface foraging, DF = deep foraging, TRA = traveling, REST = resting. The dashed lines represent 95% confidence interval (CI). The predicted values and CI were obtained using the AICcmodavg package in R. N = 6901.

We found a curvilinear relationship between dive duration and the number of subsequent breaths (i.e. short dives), which differed between activities (Fig 3). For NSF and DF, the number of breaths (i.e. short dives) increased linearly with dive duration up to a certain duration (200 and 400 seconds, respectively), after which the number of short dives remained constant (at about three and seven short dives (four and eight breaths), respectively) (Fig 3). Traveling and REST whales showed similar positive increases in number of breaths (i.e. short dives) with dive duration, however the curves levelled off already at two short dives (three breaths), after about 300 seconds dive duration (Fig 3). Although whales engaged in SFT1 and SFT2 showed curvilinear relationships between dive duration and number of subsequent breaths (i.e. short dives), the curves remained flat, and thus non-significant, over the range of dive durations observed. The intercept was higher for SFT2 than SFT1. The nonlinear relationships between the number of breaths (i.e. short dives) and dive duration were supported by the GAM, which showed similar patterns for all activity states (S1 Fig).

### Temporal scale of surfacing patterns

**Fig 4:**
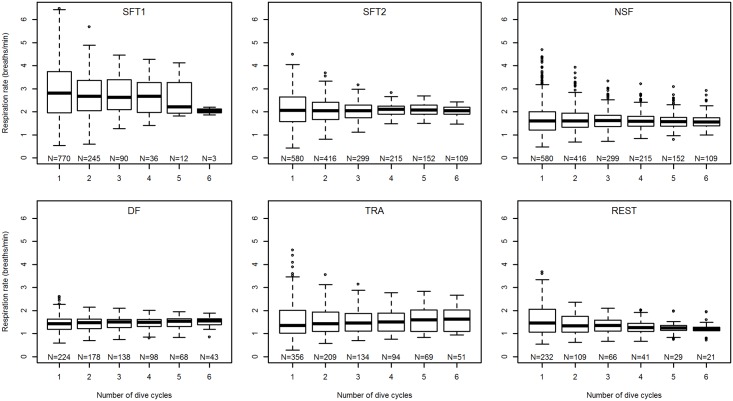
Boxplots of minke whale respiration rates over different temporal scales (dive cycles) for different activity states. SFT1 = surface feeding tactic I, SFT2 = surface feeding tactic II, NSF = near-surface foraging, DF = deep foraging, TRA = traveling, REST = resting.

Respiration rates, and hence surfacing patterns, of minke whales varied between activity states and across temporal scales (Fig 4). Non-feeding minke whales and whales foraging at depth had similar respiration rates, around 1.5 breaths min^-1^, while surface feeding whales had higher respiration rates, at about 2 and 3 breaths min^-1^ for SFT2 and SFT1, respectively. The variation in respiration rates, particularly at lower temporal scales (i.e. fewer dive cycles), was also much higher for SFT1 and SFT2 whales compared to other activities. The variance in respiration rates seemed to decrease as the number of dive cycles included in the estimate increased (Fig 4). This was particularly true for SFT1 and SFT2 whales, but also REST, which had the lowest respiration rate over several dive cycles. In contrast, NSF and DF whales showed a much smaller reduction in spread of respiration rates with increasing temporal scales, suggesting relatively lower plasticity in the surfacing patterns for these activities. Traveling whales too had a consistent spread in respiration rates, irrespective of the number of dive cycles considered. However the spread was much larger than for DF, NSF, SFT2, and even REST, suggesting a higher plasticity in surfacing pattern during TRA.

## Discussion

The aim of this study was to investigate the surfacing patterns of minke whales across their activity range on a known feeding ground. We found that no single surfacing pattern was suitable for all activity states and that minke whales adjusted their surfacing pattern to the specific activity in which they were engaged. We also found that minke whales modified their surfacing pattern both by changing the expression of their dives (i.e. density distribution), the behavioral expression, and the temporal dependence between dive types (i.e. transition probability), the behavioral process [39].

A fundamental question in regards to the surfacing patterns of aquatic air-breathing animals is whether a species prepares for a long dive [45] or recovers from it [25,29], or a combination of both [47]. In the case of minke whales, the stronger relationship between dive duration and the number of succeeding, rather than preceding, breaths (i.e. short dives) indicated that minke whales recovered rather than prepared for a long dive, similar to other rorquals [27,48].

As expected, minke whales did not show a very structured surfacing pattern during non-feeding activities, with REST having a random, and TRA a near-random pattern. Although their IBI density distributions did show a division into short and long dives, the distinction was less clear and there was a relatively larger overlap between dive types compared to other activities, the only exception being SFT1. This suggests that minke whales do not benefit much from structuring their dives during non-feeding activities. Resting animals aim to conserve energy, which is dependent on energy expenditure and not dive duration, which could explain the lack of structure in the surfacing pattern of REST whales. The respiration rate of minke whales can serve as a proxy for energy expenditure [43,49], although validation of this approach is needed using respirometry techniques and/or multi-sensor recording tags (to look at underwater movement and energy use) [50]. The respiration rate, and hence energy expenditure, of REST minke whales was the lowest for all activities, and showed relatively small variations, even at lower temporal scales. This suggests that a random surfacing pattern was sufficient to keep a low respiration rate, and hence energy expenditure, during REST. In contrast, traveling whales are likely to gain some benefits, in terms of minimizing COT, by making longer dives, since staying submerged is likely to reduce drag, and hence energy expenditure [33]. We found that TRA minke whales significantly structured their surfacing pattern, and that the number of subsequent breaths (i.e. short dives) increased with the duration of the long dives. Hence, it seems that TRA minke whales do structure their surfacing pattern to extend the duration of their long dives. However, the fact that the number of breaths (i.e. short dives) seldom exceeded two or three breaths (one or two short dives) per surfacing bout, suggests that TRA minke whales do not benefit from maximizing the duration of their long dives.

Feeding minke whales showed considerable variation in both surfacing patterns and dive expressions across activity states. As the depth of the prey layer increased between SFT1, NSF and DF, so did the duration of the long dives, resulting in a clearer segregation between long dives and short dives. Similarly, the surfacing pattern became more structured as the prey depth increased, going from a pattern where long dives were dominating, to a pattern consisting of fewer isolated long dives followed by series of short dives. This difference could reflect a shift in the ratio between foraging time (related to dive duration) and surface recovery time (related to number of short dives) as depth, and hence transit time, increased between activities [12]. This shift is likely to be a continuous process, with the surfacing pattern becoming gradually more structured as the depth of the prey layer increases between feeding bouts. However to test these hypotheses, and to model depth as a continuous rather than discrete variable (prey depth was assumed to differ between SF, NSF and DF), additional studies using animal borne multi-sensor recording tags that can measure/infer feeding events, together with information on prey patch quality (e.g. density or biomass) are needed.

For whales feeding at depth, long dives may represent searching for or ingesting prey. Differences in dive duration could represent variation in foraging behavior, prey capture efficiency, number of feeding lunges, etc. For both NSF and DF, the number of subsequent breaths (i.e. short dives) increased with dive duration up to a certain point where it levelled off. Similar non-linear relationships between surface time and dive duration have been found in blue whales (*Balaenoptera musculus*), fin whales (*Balaenoptera physalus*) and humpback whales (*Megaptera novaeangliae*) [27,48], and are likely the result of a non-linear relationship between dive duration and energy expenditure, as found in other marine mammals [29,51,52]. Baleen whales are able to reduce their energy expenditure during diving by gliding, either during the decent phase [53–55] or during the ascent phase of the dive [56], and a similar tactic could be used by minke whales to extend the duration of their long dives without having to increase energy expenditure. That minke whales sometime make shorter dives below their average (i.e. their asymptote) would suggest that other factors apart from oxygen level can determine when an animal will terminate a dive. Low prey density for example can lead marine mammals to end dives prematurely (in relation to the ADL) [15,18,27]. Since shorter dives generally have lower energy expenditure, fewer breaths will be needed to restore the oxygen levels, which can help explain the initial positive relationship between dive duration and number of subsequent breaths (i.e. short dives) in NSF and DF minke whales.

Interestingly, the two surface feeding activities showed contrasting patterns to each other, both in the expression of dive types and in surfacing patterns. While more than two thirds of the dives during SFT1 were of the long type, long dives constituted only a third of the dives performed during SFT2. With SFE occurring predominantly at the end of long dive types (83.8 and 90.3% for SFT1 and SFT2, respectively), rather than at the end of short dive types, this means that the overall feeding rate (number of SFE per time unit) was higher during SFT1 compared to SFT2. Considering respiration rates however, SFT1 had a higher respiration rate than SFT2, suggesting that the former could have a higher energy expenditure, likely as a result of the higher number of feeding lunges, which are energetically costly [57]. Further studies are hence needed to estimate the efficiency of the two surface feeding tactics. Perhaps the corralling pattern during SFT2 might be more efficient in situations when prey is relatively dispersed, whereas the surfacing pattern of SFT1 might be the better strategy when prey is already aggregated. A similar conclusion was made by Hoelzel et al. [58] who compared surface feeding strategies for minke whales on the US west coast.

The relationship between dive duration and number of subsequent breaths (i.e. short dives) showed that TRA minke whales needed about three breaths (two short dives) to recover from a 400 seconds dive, whereas NSF and DF whales needed about four and seven breaths (three and six short dives) to recover, respectively. Underwater lunge feeding has been shown to infer high energetic costs in rorquals [18,48,50,57,59], resulting in a positive linear relationship between the number of short dives and the number of feeding lunges [27]. Although minke whales incur the least energetic cost per lunge of any baleen whale due to their relatively small body size (the energetic costs of lunge feeding scale positively with body size), they also perform the highest number of lunges per dive for any lunge-feeding whale [50,59]. The relatively longer recovery times (i.e. number of succeeding short dives) for NSF and DF minke whales might therefore be caused by increased energetic costs associated with lunge feeding at depth. Considering respiration rates however, and assuming that respiration rate reflects an animal’s energy expenditure [49], it appears that the energetic costs over time are similar between TRA whales and whales foraging at depth. This makes sense since animals need to balance their oxygen level over time, irrespective of activity. The respiration rate estimates obtained over larger temporal scales corresponds well with studies on minke whales in other locations (see [21] for review).

Considering the surfacing pattern of minke whales across different temporal scales, the decreasing spread in the variance in respiration rates with increasing number of dive cycles provided quantitative evidence that minke whales can balance their oxygen level over multiple, rather than a single, dive cycle. If minke whales were balancing their oxygen level over a smaller temporal scale, the distribution of respiration rate values would have remain relatively constant with increasing temporal scales. Deviations in oxygen level over smaller temporal scales are not unusual in marine mammals [20]. Elephant seals for example incrementally accrue an oxygen debt by exceeding their estimated ADL on several consecutive dives over long periods of time, which they then repay at a later time by having extended periods of recovery [30,60,61]. Similarly, Steller sea lions (*Eumetopias jubatus*) incur an oxygen debt during diving that may not be paid back following a single dive, but across several subsequent surface series [62]. Such a surfacing pattern likely allows the animals to respond more easily to unpredictable events in their environment, which could benefit fitness over time. Although minke whales seem to exhibit some level of plasticity in surfacing patterns over smaller temporal scales, the rapid decrease in variance in respiration rates with increasing temporal scales suggest that they still balance their oxygen levels over relatively few dive cycles. The variance in respiration rates also varied between activities, with SFT1 showing the largest spread in respiration rates, even at higher temporal scales. This suggests that SFT1 whales are far from the limits of their capacity (plasticity) to alter their respiration rates in response to their environment. In contrast, DF whales showed much less variability in respiration rates, most likely reflecting a lower plasticity in surfacing pattern as a result of the physiological constraints of foraging at depth. Although our findings are compelling, sample size restricted the temporal scale of our analyses to a maximum of six dive cycles. It is known that minke whales can feed, forage and engage in other activities for longer durations than the focal follows in this study [50]. Therefore, it is possible that minke whales balance their oxygen debts or recoveries across a longer time period than the one considered in this study, which could affect the observed surfacing patterns. It would therefore be worthwhile to apply this approach to longer time series of data to find out where the oxygen debts are incurred or accounted for in minke whales.

An understanding of the surfacing pattern of aquatic air-breathing animals is important for studies on breathing and foraging ecology [12–14,63], bioenergetics (to infer energy expenditure) [43,64] and population estimates (to inform correction factors in cue-counting surveys) [65,66]. We found that the surfacing pattern of minke whales differed between activities at shorter time intervals, while over longer time scales their respiration rates even out. This study thus highlights the importance of taking into consideration the scale of observations when investigating animal behavioral patterns.

## Supporting Information

S1 Fig Back transformed number of short dives in a surfacing bout as a function of the dive duration of the preceding long dive for minke whales during different activity states. SFT1 = surface feeding tactic I, SFT2 = surface feeding tactic II, NSF = near-surface foraging, DF = deep foraging, TRA = traveling, REST = resting). A separate GAM (GAM: Nb.short~s(Dive.pre)) was fitted to each activity state. The dashed lines represent 95% confidence intervals. The smoother for dive duration was significant (*P* = 0.05) for all activity state. The sample size (N) for each activity state is shown in the upper right corner of each subfigure.
(TIFF)

S1 Dataset Minke whale focal follow data used in behavioral analyses. (TXT)

S2 Dataset Fish-finding sonar data used in prey depth analyses. (TXT)
