# Supplementary figures and images for: Structure and Dynamics of Minke Whale Surfacing Patterns in the Gulf of St. Lawrence, Canada
Source: PLoS One. 2015 May 13;10(5):e0126396. doi: 10.1371/journal.pone.0126396 (PMC4430536; doi:10.1371/journal.pone.0126396)

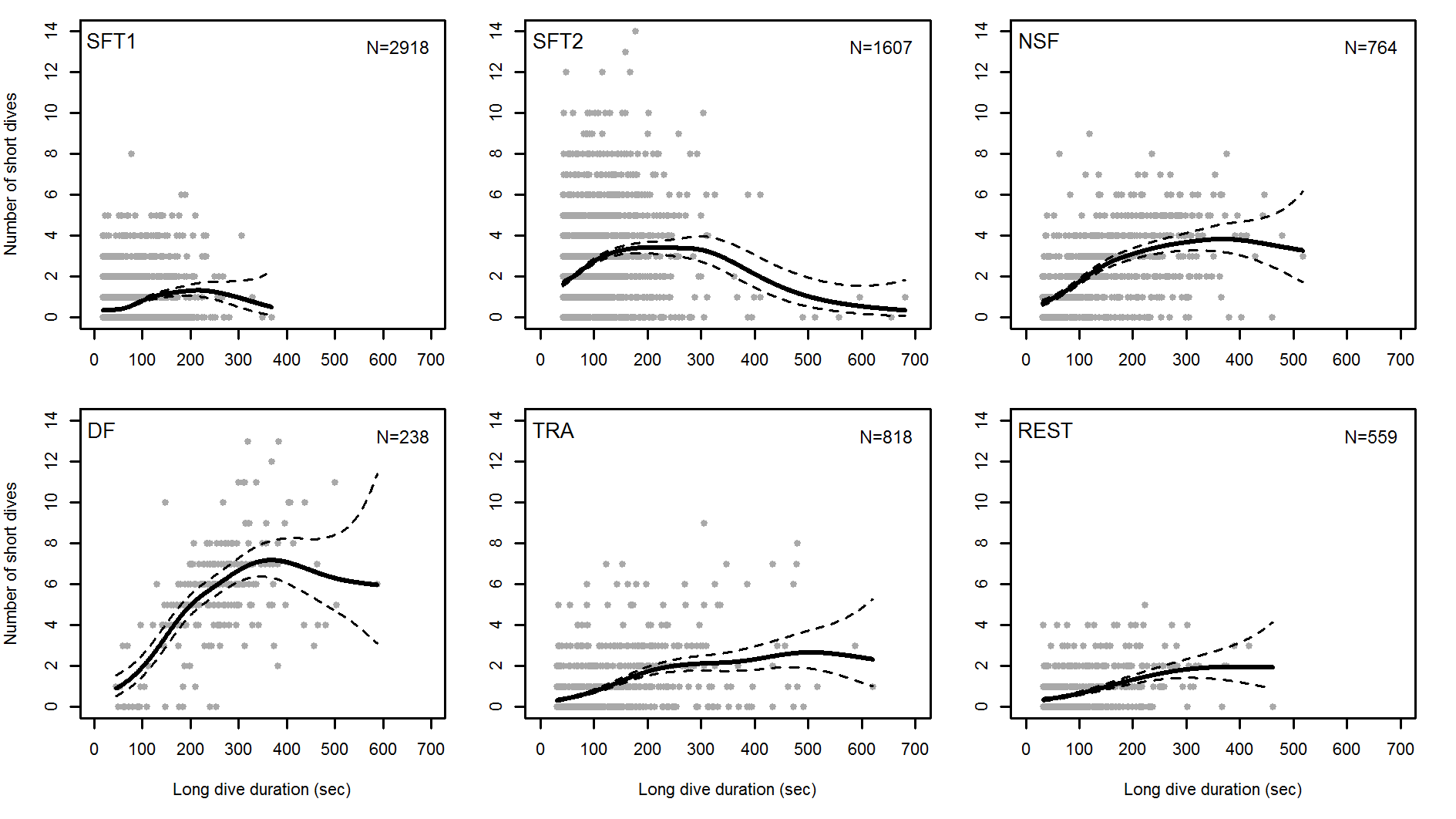

Supplement: S1 Fig — SFT1 = surface feeding tactic I, SFT2 = surface feeding tactic II, NSF = near-surface foraging, DF = deep foraging, TRA = traveling, REST = resting). A separate GAM (GAM: Nb.short~s(Dive.pre)) was fitted to each activity state. The dashed lines represent 95% confidence intervals. The smoother for dive duration was significant (P = 0.05) for all activity state. The sample size (N) for each activity state is shown in the upper right corner of each subfigure. (TIFF) [file pone.0126396.s001.tiff]
